# The meaning of additive reaction-time effects: some misconceptions

**DOI:** 10.3389/fpsyg.2013.00744

**Published:** 2013-10-17

**Authors:** Saul Sternberg

**Affiliations:** ^1^Department of Psychology, University of PennsylvaniaPhiladelphia, PA, USA

**Keywords:** stage models, additive-factors method, reaction time, RT, Stroop effect, factorial experiment, additivity

## Additive effects

**Figure 1:**
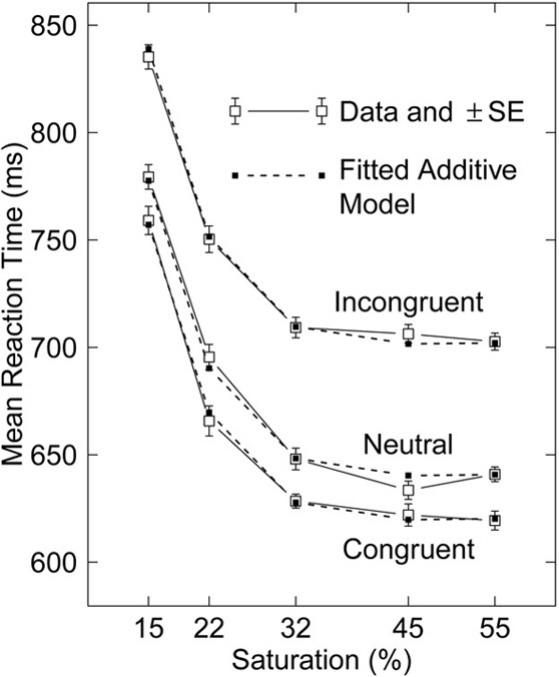
**Mean RT data from Stafford et al. (2011, Experiment 1) and fitted additive model.** Standard errors (SEs) shown (with mean 5.0 ms), which can be used to assess goodness of fit, are based on deviations from additive models plus linear interactions fitted to the 15 mean RTs from each of the 20 subjects. The SE based on the residual mean square (114 df) in an anova, after removing the effects of subject, saturation, congruence, their two-way interactions, and the interaction with subjects of the linear interaction of saturation and congruence, is 8.2 ms.

Stafford and Gurney (2011) (S&G) discuss a beautiful set of reaction-time (RT) data (Figure 1) from an experiment using the Stroop effect in which they varied the saturation of the color in which the word was printed. They found persuasive additivity of the substantial effects on mean RT of two factors: saturation of the color to be named, and congruence of the word with the color name.

Such additivity is consistent with the RT being generated by two stages (successive processes), **C**: *color discrimination*, whose duration is influenced by saturation but not congruence, and **N**: *retrieval and pronunciation of the color name*, whose duration is influenced by congruence but not saturation. (There must also be another process, **W**: *word recognition*, in which the word is identified, which perhaps ends before **N** and may operate in parallel with **C**.) S&G claim that their findings can also be explained by a model that contains a single process influenced by both factors. Because they interpret the additive factors method (AFM; Sternberg, 1969, 1984, 1998, 2001, 2011) to be associated with the idea that the additivity of the effects of two factors *implies* two stages, they conclude that the assumptions of the AFM are “untenable.”

## Inference from additivity

I believe that such additivity *supports* (increases belief in) a two-part hypothesis: processes organized serially (stages) together with selective influence of the factors on those stages, but doesn't *imply* that hypothesis (Sternberg, 2001, Table 3). If more than one model is consistent with some properties of a set of data, then selecting among models often requires a search for predictions of other properties that distinguish them, sometimes supplemented by further experiments and/or plausibility considerations. Examples are provided by Roberts and Sternberg (1993), who considered five data sets in relation to stage models together with two other models that can explain additive effects on mean RT: an “alternate pathways model” and a version of the cascade model (McClelland, 1979; Ashby, 1982). By assuming stochastic independence of stage durations to strengthen the stage models, and considering properties that depend on RT variance, they could discriminate among the alternatives. See also Schweickert et al. (2012, Ch. 6).

## Implications of alternative models

Since the AFM was introduced (Sternberg, 1969), alternatives to stage models have been developed that are consistent with means additivity (Ashby, 1982; Roberts and Sternberg, 1993; Miller et al., 1995). However, in all such models thus far, the prediction of additivity derives from functionally distinct processses plus selective influence. Thus, the existence of these alternatives does not weaken the support provided by additivity for functionally distinct and separately modifiable processes, but does weaken support for those processes being organized as stages. However, S&G's single-process model has not been shown to produce additive effects, and seems implausible.

## Are effects additive in S&G's “one-stage” model?

S&G made no attempt to fit models to their data. Even without the constraints this might impose, saturation and congruence interact systematically in their simulation of the single-process model (S&G figure 3) for which they claim additivity: for their five increasing saturation levels of 0.2, 0.4, 0.6, 0.8, and 1.0, the RT differences between incongruent and neutral conditions are 1.77, 1.10, 1.00, 1.12, and 1.47; those between neutral and congruent conditions are 2.15, 1.25, 1.04, 1.00, 1.04; both in arbitrary units. Given additivity, the differences in each set would all be equal.

## Is S&G's “one-stage” model plausible?

The single-process model for which S&G claim additive effects has “locked inputs”: to the extent that low saturation delays color discrimination it also delays word recognition. (S&G conclude that their models with unlocked inputs cannot produce the desired additivity). Because the time to recognize the word probably depends primarily on its luminance contrast (which was fixed) rather than its color saturation, a model with “locked inputs” seems implausible. Moreover, support for the idea that recognition of the word is *not* influenced by saturation is provided by the similarity of these data to the results of experiment 2 in Stafford et al. (2011), in which a white word was displayed at a different location from a patch whose color was named. If saturation had influenced the recognition of words in experiment 1, the results of the two experiments should have differed more.

## More on AFM reasoning

Others have expressed different misconceptions about the “logic” of the AFM and the nature of stage models. According to Van Zandt and Ratcliff (1995), “The use of additive mean RT's as support for a serial arrangement of subprocesses depends heavily on the assumption of selective influence …” (p. 34). Instead, the prediction of additivity should be thought of as depending on a two-part hypothesis: stages (seriality), and selective influence. Observation of additivity supports both parts, just as confirmation of a prediction from any theory supports that theory. Independent evidence for selective influence is not required.

## What is a stage model?

One misconception about the kinds of models to which the AFM is relevant is perhaps due to confusion between flow-charts (with units that are subprocessES and arrows that indicate successiveness), and block diagrams (with units that are processORS and arrows that indicate information transfer). Broadbent (1984) and Coltheart (2011) have argued that a process whose subprocesses are organized in stages cannot involve feedback because it must be implemented by a “pipeline”: an ordered set of processors through which information passes in a fixed direction from input to output. They seem to misinterpret the boxes in diagrams of stage models as spatially arranged processORS, rather than temporally arranged processES.

Stage models partition processing operations into temporally successive (and functionally distinct) components. Nothing prevents a later stage from using added information (feedback) to re-process evidence that was extracted earlier. For example, in reading a word, the initial perception of a letter might be revised, based on contextual letters; the revision process could be a processING stage in which a letter-perception processOR is re-used. Also, not all stages require stimulus representations provided by earlier stages as input: consider the serial operations that may underlie visual search for a feature conjunction, in which each comparison between the target and a displayed item can be thought of as a stage. For the distinction among three kinds of stage (completion-controlled, outcome-contingent, and data-dependent), see Sternberg (1984). Neurophysiological evidence for data-dependent stages is discussed by Schall (2003), Woodman et al. (2008), Schall et al. (2011), and Sternberg (2011, Section 3.2), and references therein. Neurophysiological evidence for completion-controlled stages is provided by Sigman and Dehaene (2008).

## Note

This article published in Frontiers in Psychology, under the section of Cognitive Science, was invited by the Chief Editor Professor Eddy Davelaar.
